# A *Burkholderia thailandensis* DedA Family Membrane Protein Is Required for Proton Motive Force Dependent Lipid A Modification

**DOI:** 10.3389/fmicb.2020.618389

**Published:** 2021-01-12

**Authors:** Pradip R. Panta, William T. Doerrler

**Affiliations:** Department of Biological Sciences, Louisiana State University, Baton Rouge, LA, United States

**Keywords:** proton motive force, membrane protein, lipid A modification, lipopolysaccharide, antibiotic resistance, colistin

## Abstract

The DedA family is a conserved membrane protein family found in most organisms. A *Burkholderia thailandensis* DedA family protein, named DbcA, is required for high-level colistin (polymyxin E) resistance, but the mechanism awaits elucidation. Modification of lipopolysaccharide lipid A with the cationic sugar aminoarabinose (Ara4N) is required for colistin resistance and is dependent upon protonmotive force (PMF) dependent transporters. *B. thailandensis* Δ*dbcA* lipid A contains only small amounts of Ara4N, likely leading to colistin sensitivity. Two *B. thailandensis* operons are required for lipid A modification with Ara4N, one needed for biosynthesis of undecaprenyl-P-Ara4N and one for transport of the lipid linked sugar and subsequent lipid A modification. Here, we directed overexpression of each *arn* operon by genomic insertion of inducible promoters. We found that overexpression of *arn* operons in Δ*dbcA* can partially, but not completely, restore Ara4N modification of lipid A and colistin resistance. Artificially increasing the PMF by lowering the pH of the growth media also increased membrane potential, amounts of Ara4N, and colistin resistance of Δ*dbcA*. In addition, the products of *arn* operons are essential for acid tolerance, suggesting a physiological function of Ara4N modification. Finally, we show that Δ*dbcA* is sensitive to bacitracin and expression of a *B. thailandensis* UppP/BacA homolog (*BTH_I1512)* can partially restore resistance to bacitracin. Expression of a different UppP/BacA homolog (*BTH_I2750)* can partially restore colistin resistance, without changing the lipid A profile. This work suggests that maintaining optimal membrane potential at slightly alkaline pH media by DbcA is responsible for proper modification of lipid A by Ara4N and provides evidence of lipid A modification-dependent and -independent mechanisms of colistin resistance in *B. thailandensis.*

## Introduction

Colistin (polymyxin E) is a polycationic and amphiphilic peptide which is a last resort antibiotic for treatment of many Gram-negative bacterial infections. Discovered in 1947 (Ainsworth et al., 1947), and isolated in 1949 from *Bacillus polymyxa* broth (Koyama, 1950), colistin was not widely used due to nephrotoxicity and neurotoxicity after intravenous administration (Koch-Weser et al., 1970). However, clinical use of colistin is increasing due to limited alternative therapeutic options (Li et al., 2006). With increased colistin use, the emergence of colistin resistance has been documented in several human pathogens, such as *K. pneumoniae* (Bratu et al., 2005; Antoniadou et al., 2007; Farzana et al., 2020), *Salmonella enterica* serovar Newport (Elbediwi et al., 2020), and *Acinetobacter baumannii* (Marano et al., 2020; Papathanakos et al., 2020). More worrisome is the recent global spread of mobilized colistin resistance (*mcr*) genes (Elbediwi et al., 2019). Therefore, understanding the molecular mechanism of colistin resistance is of utmost importance at times when the antibiotic discovery pipeline is drying up.

Species belonging to the genus *Burkholderia* are intrinsically colistin resistant with minimal inhibitory concentrations (MIC) often exceeding 500 μg/ml (Loutet and Valvano, 2011). The molecular mechanisms behind this intrinsic colistin resistance of *Burkholderia* spp. is not yet fully understood. Electrostatic interaction of colistin with negatively charged lipopolysaccharide (LPS) lipid A in the outer membrane of Gram-negative bacteria and disruption of outer membrane is understood as the initial mode of action (Hancock and Chapple, 1999; Velkov et al., 2010). A common mechanism of colistin resistance among many Gram-negative bacteria is expression of biosynthetic pathways that result in the modification of LPS lipid A with cationic substituents aminoarabinose (Ara4N) or phosphoethanolamine (Raetz et al., 2007; Needham and Trent, 2013; Olaitan et al., 2014; Simpson and Trent, 2019). This reduces the electrostatic attraction between colistin and lipid A and thus results in colistin resistance.

The modification of lipid A with Ara4N begins with the enzymatic conversion of UDP-glucose to UDP-glucuronic acid in the cytoplasm by UDP-Glc dehydrogenase (*ugd*) (Breazeale et al., 2002). Then, several proteins ArnA, ArnB, ArnC, and ArnD convert UDP-glucuronic acid to undecaprenyl-P-Ara4N on the cytoplasmic leaflet of the inner membrane (Noland et al., 2002; Breazeale et al., 2003, 2005; Williams et al., 2005). Putative EmrE like transporters, ArnE and ArnF, flip undecaprenyl-P-Ara4N from the cytosolic leaflet to the periplasmic leaflet of the inner membrane (Yan et al., 2007) where another membrane protein ArnT transfers Ara4N to the lipid A (Trent et al., 2001). This is followed by the transport to the outer membrane by lipopolysaccharide transport pathway (Lpt) (Li et al., 2019; Owens et al., 2019). Previously, we found that a DedA family membrane protein, DbcA (DedA of *Burkholderia* required for colistin resistance) is required for lipid A modification with Ara4N and high level colistin resistance in *Burkholderia thailandensis* (Panta et al., 2019). The molecular mechanism behind this is unclear.

The DedA family is a highly conserved membrane protein family present within all three domains of life (Doerrler et al., 2013). There are currently 25,081 individual sequences in the protein database across 7172 species belonging to the “SNARE-associated PF09335” family of proteins (PFAM 33.1). An *E. coli* DedA family mutant (Δ*yqjA*, Δ*yghB*) was found to have defects in membrane potential and cell division, induction of extracytoplasmic stress responses, sensitivity to alkaline pH, temperature sensitivity, and sensitivity to several classes of antibiotics and membrane penetrating dyes (Thompkins et al., 2008; Sikdar and Doerrler, 2010; Sikdar et al., 2013; Kumar and Doerrler, 2014, 2015). Recently, a human DedA protein, TMEM41B, has been characterized as a novel ER-localized regulator of autophagosome formation and lipid mobilization (Moretti et al., 2018; Morita et al., 2018; Shoemaker et al., 2019).

**FIGURE 1:**
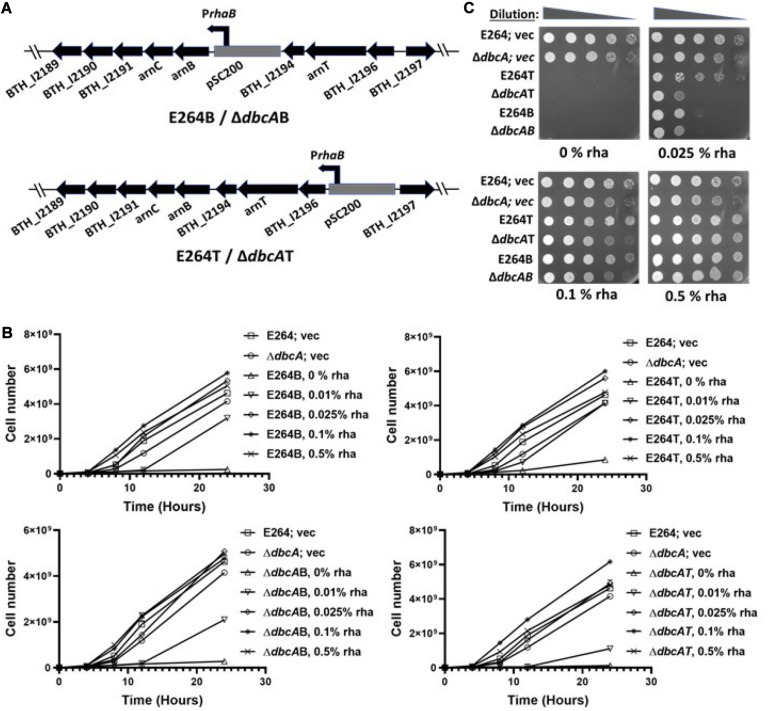
Generation and characterization of Ara4N synthesis and transport conditional mutants. **(A)** Genetic organization of *arn* gene clusters and flanking regions of *B. thailandensis* E264. The gray box represents the plasmid, pSC200 with an inducible rhamnose promoter *(PrhaB).* E264B/Δ*dbcA*B (B for biosynthesis) represents a strain of E264 or Δ*dbcA*:FRT with pSC200 insertion in front of the putative *arn* biosynthesis transcriptional unit. E264T/Δ*dbcA*T (T for transport) represents a strain of E264 or Δ*dbcA*:FRT with pSC200 insertion in front of the putative *arn* transport transcriptional unit. **(B)** Growth rate of E264; vec, Δ*dbcA*; vec, E264B, E264T, Δ*dbcA*B, and Δ*dbcA*T at times 0, 4, 8, 12, and 24 h in LB liquid media containing the indicated concentrations of rhamnose. The graph was created through GraphPad Prism 8.4.3. The error bars indicate standard deviations of three biological replicates. **(C)** Spot assay of E264; vec, Δ*dbcA*; vec, E264B, E264T, Δ*dbcA*B, and Δ*dbcA*T on LB agar media. 1:10 dilutions of indicated strains from 2nd overnight culture grown without any rhamnose were spotted on LB, 100 Tmp, and different amounts of rhamnose as indicated. Plates were analyzed after 24 h of growth at 37°C.

*B. thailandensis* DbcA has been shown to be required for extreme colistin resistance, probably due to compromised proton motive force (PMF) (Panta et al., 2019). We proposed a model which suggests that the lower membrane potential observed in Δ*dbcA* is responsible for inefficient export of undecaprenyl-P-Ara4N by EmrE-like transporter BTH_I2194 (ArnEF homolog). We reasoned that overexpression of the *arn* genes in Δ*dbcA* could compensate colistin hypersensitivity of Δ*dbcA*. Based on Prokaryotic Operon Database (ProOpDB), putative *arn* genes in *B. thailandensis* are organized in two transcriptional units (Figure 1A), consistent with the finding in *Burkholderia cenocepacia* (Ortega et al., 2007; Taboada et al., 2012). Lipid A modification with Ara4N is essential for bacterial viability, LPS export, proper assembly of outer membrane, and intrinsic resistance to polymyxin B in *B. cenocepacia* (Ortega et al., 2007; Hamad et al., 2012). Absence of transposon mutants of aminoarabinose synthesis and transport genes suggest that *arn* genes are also essential in *B. thailandensis* (Gallagher et al., 2013). We therefore used a conditional mutagenesis technique where an inducible rhamnose promoter is inserted into the chromosome to drive the expression of targeted genes (Ortega et al., 2007). This enabled us to directly control the expression of *arn* gene clusters and study the role of lipid A modification in colistin resistance in both WT and Δ*dbcA* strains. We show evidence for a link between pH and membrane potential and how lipid A modification and colistin resistance in *B. thailandensis* is linked to pH homeostasis.

## Materials and Methods

### Culture Conditions

The bacterial strains and plasmids used in this study are listed in Supplementary Table S1. *E. coli* and *B. thailandensis* were grown in LB medium (1% tryptone, 0.5% yeast extract, and 1% NaCl) unless otherwise indicated. Colistin (Col), trimethoprim (Tmp), rhamnose (rha), 2,6 diaminopimelic acid (DAP), bacitracin (Bac) were purchased from VWR or MilliporeSigma. Cultures were grown at 37°C shaking at 225 rpm.

E264B, E264T, Δ*dbcA*B, and Δ*dbcA*T strains were inoculated in 5 ml LB, 100 Tmp, and 0.1% rha and grown for ∼24 h. One milliliter culture was removed, washed with LB, and resuspended in fresh LB. 2.5 × 10^7^ cells from all the strains were then inoculated in 5 ml fresh LB, 100 Tmp without any rhamnose and grown for 24 h. One milliliter of this O/N cultures without any rhamnose were again removed, washed with LB, and resuspended in fresh LB. Then 2.5 × 10^7^ cells were inoculated in 50 ml LB, 100 Tmp with different concentrations of rhamnose and grown at 37°C and 225 rpm. Cell density was measured by optical density at 600 nm.

### Generation of *Burkholderia thailandensis arn* Conditional Mutants

Creation of *B. thailandensis arn* conditional mutants was carried out by inserting the plasmid pSC200 upstream of either the *arn* biosynthetic cluster or *arn* transport cluster (Figure 1A) so that the expression of these operons could be regulated by the plasmid-borne inducible rhamnose promoter as previously described (Ortega et al., 2007; Wagley et al., 2014). PCR primers are listed in Supplementary Table S2. The plasmid, pSC200 was a generous gift by Dr. Miguel A. Valvano (Queen’s University Belfast, United Kingdom). Two primers FWAra4NB and REVAra4NB were used to PCR amplify ∼290 bp portion of BTH_I2193 (*arnB*), the first gene in *arn* biosynthetic cluster (Supplementary Figure S1). Primers FWAra4NT and REVAra4NT were used to amplify ∼330 bp portion of BTH_I2196, the first gene in *arn* transport cluster (Supplementary Figure S1). The PCR fragments were ligated to pSC200 using Nde1 and Xba1 restriction sites, upstream of the rhamnose promoter, transformed into *E. coli* XL1-blue cells, and selected on LB agar with 50 μg/ml Tmp. The plasmids pSC200Ara4NB and pSC200Ara4NT were further transformed into *E. coli* RHO3 by electroporation (Tu et al., 2016). *E. coli* RHO3 is a generous gift of Dr. Erin C. Garcia (Department of Microbiology, Immunology, and Molecular Genetics, University of Kentucky College of Medicine). *E. coli* RHO3 is a donor strain sensitive to all commonly used antibiotics and its DAP auxotrophy makes it easy to counter select on rich media by the exclusion of DAP from the selection media (Lopez et al., 2009). RHO3 transformants were then conjugated with *B. thailandensis* E264 and Δ*dbcA:*FRT (Garcia, 2017). Colonies were screened on LB agar with 100 μg/ml Tmp and 0.25% rhamnose. The Tmp^R^ transconjugants were further grown in Tmp and rhamnose and their extracted genomic DNA was used to confirm the integration of the plasmids pSC200Ara4NB or pSC200Ara4NT in E264 or Δ*dbcA:*FRT by using primers ConfirmAra4NB and ConfirmAra4NT as shown in the Supplementary Figure S1. The inability of these strains to grow on LB plates without rhamnose further confirmed the success of generation of conditional arn mutants in E264 and Δ*dbcA:*FRT. We named these strains E264B, E264T, Δ*dbcA*B, and Δ*dbcA*T as shown in Figure 1A.

### Susceptibility Assays

For testing the susceptibility on solid medium, *B. thailandensis* strains were grown for 18–24 h in fresh LB broth supplemented with 100 μg/ml Tmp and rhamnose if required. 1 ml of an overnight culture was washed 1x with fresh LB, and resuspended with 1 ml fresh LB. The cell concentration was adjusted to 3 × 10^8^ cells/ml. Five microliters of serially log_10_-diluted cells was then spotted on LB agar plates containing various concentrations of antibiotics and rhamnose as required. Growth was analyzed after incubation for 24–72 h at 37°C as indicated in figure captions. The colistin MIC was measured using Liofilchem^®^ MIC Test Strips (Liofilchem, Inc.). Overnight cultures were adjusted to 1 × 10^8^ cells/ml as described above and a sterile swab was used to create a lawn of cells. Then the MIC strip was applied to the plates and the growth was evaluated after 24–48 h at 37°C. All experiments were repeated at least three times.

### Measurement of Membrane Potential

Measurement of membrane potential was done using JC-1 dye as previously described (Panta et al., 2019). Briefly, 1 ml of overnight cultures grown in LB media broth with appropriate antibiotic and reagents were washed with fresh LB and resuspended in 1 ml LB media. 2.5 × 10^7^ cells were inoculated in 25 ml of fresh LB broth in 250 ml flask and grown for about 7 h. Then ∼6 × 10^8^ cells were taken from each culture, washed with permeabilization buffer, PB (10 mM Tris, pH 7.5, 1 mM EDTA, 10 mM glucose) and finally resuspended in PB buffer. Three micromolar of JC-1 dye was added, incubated in the dark at 37°C for 30 min. Cells were washed and resuspended in PB buffer and fluorescence measurements were carried out using a JASCO FP-6300 spectrofluorometer. Membrane potential is determined by the ratio of red (595 nm) to green (530 nm) fluorescence with excitation of 488 nm.

### Analysis of Lipid A by TLC

Lipid A analysis was performed as described (Doerrler et al., 2004). Cells were labeled with 5 μCi/ml of ^32^P in 2 ml of LB broth at an OD_600_ of ∼0.8 for 30 min shaking at 37°C. The cells were collected using a centrifuge and washed with PBS, pH 7.4. To extract the ^32^P-labeled phospholipids, the cell pellet was resuspended in 1 ml of a single-phase Bligh/Dyer mixture, consisting of chloroform/methanol/water (1:2:0.8, v/v). After mixing and incubating for 30 min at room temperature, the insoluble material was removed by centrifugation, and the supernatant containing the ^32^P-labeled phospholipids was removed. The insoluble residue, which contains the ^32^P-labeled lipopolysaccharide, was subjected to hydrolysis at 100°C in 0.2 ml 12.5 mM sodium acetate buffer, pH 4.5, in the presence of 1% SDS to cleave the Kdo-lipid A linkage. The released ^32^P-labeled lipid A species were extracted by the addition of 0.25 ml CHCl_3_ and 0.5 ml MeOH. Following centrifugation, the supernatant was transferred to new tube to which an additional 0.25 ml CHCl_3_ and 0.25 ml water was added. The lower phase was washed with fresh upper phase and dried in a speed-vac. Dried material was dissolved in a small volume of chloroform/methanol 4:1 and spotted onto a Silica Gel 60 thin layer chromatography (TLC) plate which was developed in the solvent chloroform, pyridine, 88% formic acid, water (50:50:16:5, v/v). The plate was dried and exposed to a Phosphor-Imager screen overnight to visualize the ^32^P-lipid A species.

### Quantification of Lipid A Species

A TLC autoradiograph with the resolved lipid A species obtained from Phosphorimager equipped with IQMac software was further analyzed through ImageQuant Tools Version 2.2 for lipid A species quantification. Lipid A species modified with 0, 1, and 2 Ara4N were assigned as “0,” “1,” and “2,” respectively. The intensity of each assigned species was quantified and values for species 0, 1, and 2 were added to get the total amount of lipid A species. Percentage of each species was calculated using the following equations:

%species0=[0/(0+1+2)]×100

%species1=[1/(0+1+2)]×100

%species2=[2/(0+1+2)]×100

### Transformation and Complementation Analysis

Transformation of *E. coli* was done using a heat shock method (Froger and Hall, 2007). Transformation of *B. thailandensis* was carried out using conjugation (Garcia, 2017). Briefly, recipient *B. thailandensis* and donor *E. coli* RHO3 strains carrying Tmp^R^ plasmid(s) to be transferred were grown on LB and LB with 50 μg/ml Tmp and 200 μg/ml DAP, respectively. After 48 h of incubation at 37°C, using a sterile swab, several large colonies of both recipient and donor strains were inoculated by thoroughly spreading them on LB plates supplemented with DAP and incubated at 37°C. Control plates with recipient cells only, and donor cells only were also inoculated in a similar way. After ∼18 h of incubation, a loopful of bacterial cells from conjugation plates and control plates were streaked on LB with 100 μg/ml Tmp plates for selection. DAP was excluded on these plates to select against the donor strain, RHO3. After 48 h of incubation at 37°C, isolated Tmp^R^ colonies were used for colony PCR using plasmid specific primers (ConfirmpSCrhaB2FW and ConfirmpSCrhaB2REV) to confirm the introduction of Tmp^R^ plasmids into *Burkholderia* recipient strains.

For complementation analysis, pSCrhaB2 plasmid was used (Cardona and Valvano, 2005). *BTH_I1512* was PCR amplified using FWBTH_I1512 and REVBTH_I1512 primers. *BTH_I2750* was amplified using FWBTH_I2750 and REVBTH_I2750 primers. These fragments were then ligated into pSCrhaB2 using Nde1 and *Hin*dIII separately giving pSCuppP1 and pSCuppP2. RHO3 strains were then transformed with these plasmids and introduced into E264 and Δ*dbcA*:FRT by conjugation.

### Statistical Analyses

Values represent mean ± standard deviation of three independent determinations and statistical significance was calculated by unpaired Student’s *t*-test using GraphPad Prism 8.4.3. ^∗∗∗^*p* < 0.001, ^∗∗^*p* < 0.01. All experiments were repeated at least three times.

## Results

### Growth Curve for *arn* Conditional Mutants of E264 and Δ*dbcA*

*Arn* genes in *B. thailandensis* are organized in two transcriptional units as shown in Figure 1A (Ortega et al., 2007; Taboada et al., 2012). One transcriptional unit encodes the gene products for synthesis of undecaprenyl-P-Ara4N; BTH_I2193 (41% amino acid identity with *E. coli* K-12 ArnB), BTH_I2192 (48% identity with ArnC), BTH_I2191, BTH_I2190 (28% identity with ArnA), BTH_I2189 (36% identity with ArnD). Another transcriptional unit encodes *arn* transport gene products- BTH_I2196, *BTH_I2195* (23% identity with ArnT), and *BTH_I2194* (27% identity with ArnE/ArnF) (Figure 1A). Alignments were performed using Needleman-Wunsch alignment (Altschul et al., 1997, 2005).

In our study, we used the plasmid pSC200 to insert an inducible rhamnose promoter upstream of *arn* synthesis operon (creating strains E264B and Δ*dbcA*B) or upstream of *arn* transport operon (creating strains E264T and Δ*dbcA*T) as shown in Figure 1A. The plasmid pSC200 has multiple-cloning site, ori_*R*__6__K_, and mob genes from pGpΩTp and P*rhaB* rhamnose-inducible promoter, *rhaR*, *rhaS*, and the *dhfr* cassette from pSCrhaB2 (Ortega et al., 2007). Ori_*R*__6__k_ requires pir protein in trans for replication which is not present in *B. thailandensis.* This allows the plasmid to be integrated into the chromosome at a specific site by homologous recombination. The insertions were confirmed by PCR (Supplementary Figure S1) and DNA sequencing.

The strains E264B, Δ*dbcA*B, E264T, and Δ*dbcA*T were grown in LB broth with different concentrations of rhamnose for 24 h and the cell number was measured at different times. None of the strains grew without rhamnose except controls E264; vec and Δ*dbcA*; vec (Figure 1B). For other strains, growth was rhamnose dependent, reaching the highest cell numbers at 0.1 and 0.5% rhamnose (Figure 1B). These strains were also grown on plates with and without rhamnose (Figure 1C) and the results were consistent with growth in liquid media, all suggesting that *arn* genes in *B. thailandensis* are essential, consistent with previous findings (Ortega et al., 2007). We note that E264B, Δ*dbcA*B, E264T, and Δ*dbcA*T struggle to grow in 0.025% LB agar plates whereas they grow better on LB broth as shown in Figure 1B.

### Overexpression of *arn* Operons Increases the Abundance of Lipid A Doubly Modified With Ara4N

**FIGURE 2:**
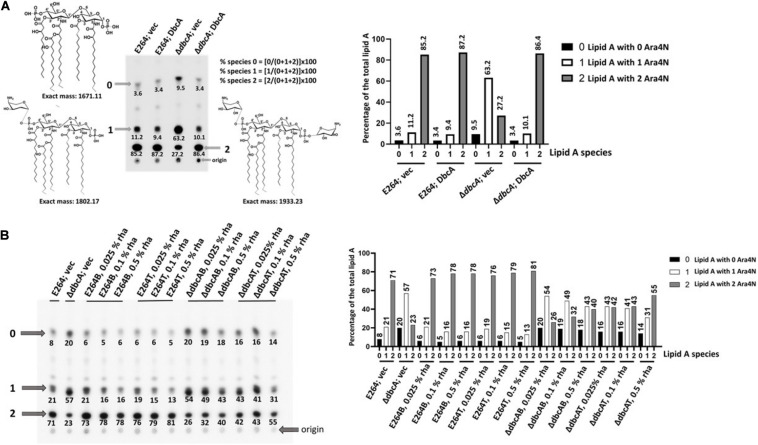
Lipid A analysis by TLC. **(A)** Lower abundance of lipid A modified with two Ara4N (species 2) in Δ*dbcA.* Lipid A was extracted from the indicated strains following labeling with ^32^P and resolved using thin layer chromatography. Analysis was conducted using a Phosphorimager equipped with IQMac software. Indicated structures were ascertained from comparison with our MS data (Panta et al., 2019). Species “2”, “1,” and “0” correspond to pentaacylated lipid A modified with two, one, or zero Ara4N, respectively. Numbers below each spot correspond to the percentage of that species of the total signal of species “2”, “1,” and “0.” **(B)** Analysis of species 2 (pentaacylated lipid A modified with two Ara4N) with different concentrations of rhamnose in E264B, E264T, Δ*dbcA*B, and Δ*dbcA*T strains. E264; vec and Δ*dbcA*; vec were included as controls. A bar graph is also included for clarity.

We previously showed using mass spectrometry that Δ*dbcA* had lower amounts of lipid A modified with Ara4N (Panta et al., 2019). Species “2,” “1,” and “0” marked by arrows in Figure 2A represent lipid A with 2 Ara4N, 1 Ara4N, and no Ara4N respectively (Figure 2A). Only 27% of the total lipid A from Δ*dbcA* is doubly modified (species 2), compared to 85–90% for the other strains (Figure 2A). To investigate whether the overexpression of *arn* operons can increase the abundance of species 2 lipid A in Δ*dbcA*, we analyzed the lipid A profile by ^32^P labeling and TLC for each strain at different rhamnose concentrations. We found that the level of species 2 increased as rhamnose concentration increased in Δ*dbcA*B, and Δ*dbcA*T strains (Figure 2B). At 0.025% and 0.5% rhamnose, species 2 for Δ*dbcA*B increased from 26% of total lipid A to 40%. For Δ*dbcA*T, species 2 increased from 40% to 55% of the total lipid A as rhamnose concentration increased from 0.025% to 0.5%. This trend is consistent with the idea that overexpressing the transport pathways is more efficient in increasing species 2 in Δ*dbcA*.

### Overexpression of *arn* Operons Can Partially Complement Colistin Sensitivity

**FIGURE 3:**
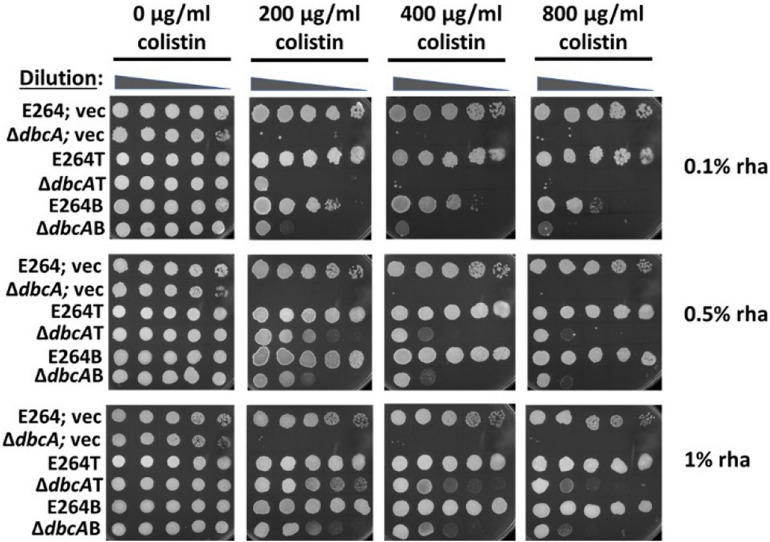
Partial complementation of colistin sensitivity in Δ*dbcAB* and Δ*dbcA*T. All the strains were grown similarly as described in Figure 1B. 1:10 dilutions were spotted on LB, 100 Tmp plates with different amount of colistin and rhamnose as indicated. Plates were analyzed after 72 h of incubation at 37°C. Higher concentrations of rhamnose are used for this experiment because the rhamnose promoter is integrated in the chromosome and *arn* genes are transcribed through this promoter as a single copy in compared to multicopy expression plasmids used elsewhere.

To investigate whether overexpression of *arn* operons can compensate colistin hypersensitivity in Δ*dbcA*, we measured growth on solid plates using a spotting technique with 0.1, 0.5, and 1% rhamnose and different concentrations of colistin (Figure 3). After ∼72 h of growth, Δ*dbcA*T and Δ*dbcA*B were able to grow much better than Δ*dbcA* in the presence of colistin. Δ*dbcA*T grew slightly better than Δ*dbcA*B in presence of colistin suggesting that overexpression of the transport operon rescues Δ*dbcA* colistin sensitivity better than overexpression of the biosynthesis operon, consistent with the lipid A profile in Figure 2B. It should also be noted that 0.5% rhamnose improved growth of the parent strains E264B and E264T compared to E264; vec in the presence of high concentrations of colistin (Figure 3). One possibility is that overexpression of these operons may improve the rate of lipid A modification with Ara4N, leading to better growth in the presence of these high concentrations of colistin. Despite this, it is likely the effects we are seeing in the compared Δ*dbcA* strains are real.

### pH Dependence of Colistin Sensitivity, Lipid A Modifications, and Membrane Potential in Δ*dbcA*

**FIGURE 4:**
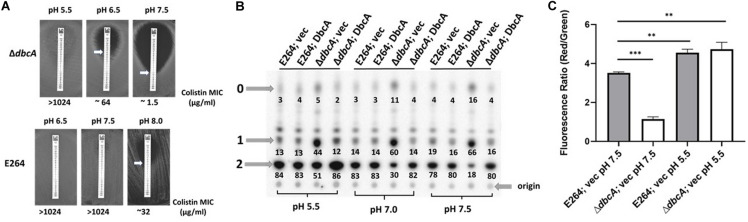
pH dependent colistin sensitivity, lipid A modification and membrane potential in *B. thailandensis*. **(A)** Minimal inhibitory concentration (MIC) was determined for indicated strains using colistin E-test strips on LB, 100 Tmp plates buffered with 100 mM of MES for pH 5.5 and 6.5, and 70 mM BIS-TRIS propane (BTP) buffer for pH 7.5 and 8.0. Approximate MIC is denoted by white arrows. Plates were analyzed after 48 h. **(B)** Analysis of lipid A at different pH media adjusted with either MES or BTP as described above. Lipid A was analyzed as previously described in Figure 2. Numbers below each spot correspond to the percentage of that species of the total signal of species “2”, “1,” and “0.” A “percentage of the total lipid A” bar graph for this figure is shown in Supplementary Figure S3. **(C)** Assessment of membrane potential (Δψ) of E264; vec and Δ*dbcA*; vec at different pH media using JC-1 dye represented as the red (595 nm)/green (530 nm) ratio. 2.5 × 10^7^ cells from an overnight culture were inoculated in 25 ml LB, 100 Tmp, pH 5.5 (MES adjusted) or pH 7.5 (BTP adjusted). Each experiment was repeated three times. Bars represent mean ± *SD* of three independent determinations and statistical significance was calculated by unpaired Student’s *t*-test using GraphPad Prism 8.4.3 ***p < 0.001, **p < 0.01.

Previous reports have shown that cell division defects, antibiotic sensitivity, and temperature sensitivity occur as a result of deletion of two genes belonging to the *E. coli* DedA protein family (*yqjA* and *yghB*) (Thompkins et al., 2008; Sikdar and Doerrler, 2010; Kumar and Doerrler, 2014). These phenotypes were rescued by lowering the pH of the media (Sikdar et al., 2013; Kumar and Doerrler, 2014). To test if lowering the pH of the media can rescue colistin sensitivity of Δ*dbcA*, we measured the colistin MIC when grown in media of different pH. Lowering the pH of the media was able to increase the colistin MIC in Δ*dbcA*; vec (Figure 4A, upper) from 1.5 at pH 7.5 to 64 at pH 6.5 to more than 1,024 at pH 5.5, all consistent with low pH complementation of different phenotypes of the *E. coli* mutant. In fact, colistin sensitivity of wild type *B. thailandensis* is dependent on the external pH of the media. The colistin MIC of strain E264 dropped to around 32 at pH 8.0 (Figure 4A, lower), which suggests that colistin resistance itself is linked to pH homeostasis in *B. thailandensis*. The MIC was also measured using the broth microdilution method (Supplementary Figure S2) which is consistent with the colistin E-test strips on solid media (Figure 4A).

To investigate whether complementation of colistin sensitivity of Δ*dbcA* at low pH is due to lipid A modification by Ara4N, we looked at the lipid A profile of all 4 strains grown at different pH. To our surprise, the level of species 2 in Δ*dbcA*; vec increased from 18% at pH 7.5 to 30% at pH 7.0 to 51% at pH 5.5 media (Figure 4B), consistent with the increase in colistin MIC from pH 7.5 to pH 5.5 in Δ*dbcA*; vec (Figure 4A, upper). However, the level of species 2 in E264 remains relatively constant as the pH was altered (Figure 4B), suggesting that DbcA helps to maintain lipid A modifications with Ara4N as external pH increases.

We previously proposed that the membrane potential plays an important role in lipid A modification and colistin resistance (Panta et al., 2019). Colistin sensitivity of Δ*dbcA* is rescued by simply growing Δ*dbcA* in acidic pH media (Figure 4A). Increased Ara4N modification of lipid A at acidic pH could be due to possible compensation of lower membrane potential at acidic pH media due to increased ΔpH component of the PMF. We measured membrane potential at different pH media and found that lower membrane potential of Δ*dbcA*; vec was corrected at pH 5.5 (Figure 4C). This suggests that the restoration of lower membrane potential at pH 5.5 is responsible for increasing the species 2 in Δ*dbcA*; vec (Figure 4B) and increasing the colistin MIC in Δ*dbcA*; vec (Figure 4A, upper). Another interesting observation was that the membrane potential of wild type E264 at pH 7.5 was significantly lower than that at pH 5.5 (Figure 4C).

### Products of *arn* Operons Are Essential for Acid Tolerance in *Burkholderia thailandensis*

**FIGURE 5:**
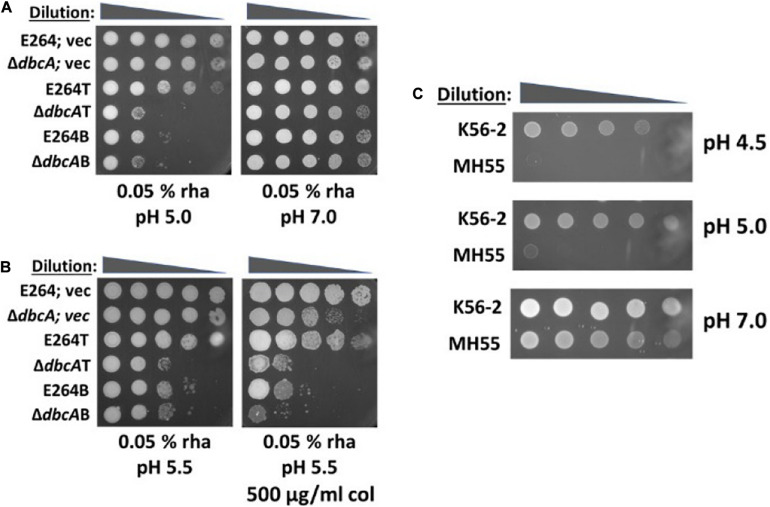
A link between low pH and lipid A modification with Ara4N. **(A)** Lipid A modification with Ara4N is essential for acid tolerance. 1:10 dilutions of indicated strains were spotted on different pH media, LB and 100 Tmp agar plates with indicated concentration of rhamnose. Plates were buffered with either MES or BTP. The plates were analyzed after 72 h of incubation at 37°C. **(B)** Low pH complementation of colistin sensitivity of Δ*dbcA* depends on the expression of *arn* operons. 1:10 dilutions of indicated strains were spotted on LB, 100 Tmp, pH 5.5 with 0.05% of rhamnose and colistin as indicated. The plates were analyzed after 72 h of incubation at 37°C. **(C)** *Burkholderia cenocepacia* ArnT suppressor mutant strain, MH-55 is sensitive to acid stress. 10-fold dilution of 3 × 10^8^ cells from overnight cultures of K-56 and MH-55 strains were spotted on LB plates at different pH media plates adjusted with either MES or BTP.

Growth in mild acid (pH 5.8) has been shown to upregulate Salmonella *arn* genes via crosstalk between PhoP/PhoQ and PmrA/PmrB two component systems contributing to polymyxin B resistance (Perez and Groisman, 2007; Chen and Groisman, 2013). It is possible that lipid A modification could be induced in low pH independent of DbcA and compensate colistin sensitivity of Δ*dbcA*. First, we tested whether *arn* operons are essential at low pH. We observed that E264B, Δ*dbcA*B, and Δ*dbcA*T strains all struggled at pH 5.0 when expression was constrained with 0.05% rhamnose induction (Figure 5A). However, at pH 7.0 media plate, all strains grew better (Figure 5A), suggesting that *arn* operons are in fact essential for extreme acid tolerance. Second, to examine if complementation of colistin sensitivity of Δ*dbcA* at low pH is through the expression of *arn* operons, we restrained the expression of *arn* operons by using 0.05% rhamnose in growth media and looked at the colistin sensitivity at pH 5.5. While lowering the pH of the media was able to rescue colistin sensitivity of Δ*dbcA*; vec, Δ*dbcA*T, and Δ*dbcA*B still remained sensitive to colistin with 0.05% of rhamnose (Figure 5B). E264B also struggles in presence of colistin at pH 5.5 with 0.05% rhamnose, all suggesting that *arn* gene products are essential for acid tolerance. To further support this, we used *Burkholderia cenocepacia ArnT* suppressor mutant strain, MH55 to test if lipid A modification by *ArnT* is essential at acidic pH. *B. cenocepacia* strains K56-2, and MH55 were generous gifts of Dr. Miguel A. Valvano (Queen’s University Belfast, United Kingdom). MH55 is a strain lacking Ara4N transferase *ArnT* harboring suppressor mutation in *lptG*, which has been shown to lack Ara4N both in the lipid A and LPS core oligosaccharide (Hamad et al., 2012). MH55 was sensitive to pH 4.5 and pH 5.5 compared to its parent strain K56-2 (Figure 5C) consistent with an essential role for Ara4N for adaptation to acidic environments.

### DbcA Is Required for Bacitracin Resistance

**FIGURE 6:**
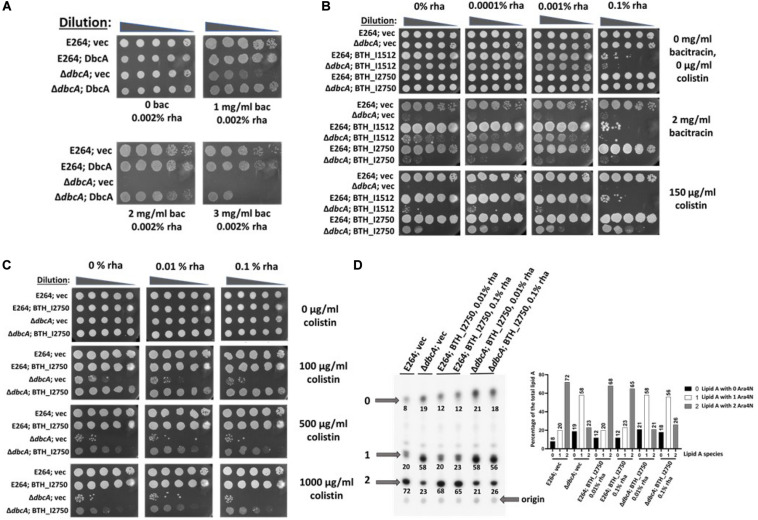
Complementation of bacitracin and colistin sensitivity of Δ*dbcA*; vec by two *B. thailandensis uppP* homologs (*Bth_I1512* and *Bth_I2750*) **(A)** Δ*dbcA* is sensitive to bacitracin. 10-fold dilution of cells from overnight cultures of indicated strains were spotted on LB, 100 Tmp, 0.002% rha and different amount of bacitracin. **(B)** Complementation of bacitracin and colistin sensitivity of Δ*dbcA* by *Bth_I1512* and *Bth_I2750*, respectively. **(C)** Partial complementation of Δ*dbcA* colistin sensitivity by *Bth_I2750.* Here, we used a multicopy expression plasmid that requires a lower concentration of rhamnose for adequate expression, compared to the integrated construct used in Figure 3. **(D)** Lipid A analysis by TLC with overexpressed *Bth_I2750* in E264 and Δ*dbcA*. Lipid A was extracted and analyzed for indicated strains. Species 2 was compared for all the strains. A “percentage of the total lipid A” bar graph is also shown on the right-hand side for more clarity.

Previously, we proposed that lower membrane potential observed in Δ*dbcA* might affect the transport activity of undecaprenyl pyrophosphate [C_55_P(P)] phosphatase UppP/BacA (Panta et al., 2019), which bears similarity to MdfA, a prototypical H^+^-coupled multidrug transporter, known to utilize PMF (Fluman et al., 2012). UppA/BacA has been shown to be involved in bacitracin resistance and is the C_55_P(P) phosphatase in *E. coli* (El Ghachi et al., 2004). To examine whether UppP/BacA plays a role in efficient lipid A modification and colistin resistance, we first tested if Δ*dbcA* is sensitive to bacitracin. Bacitracin is an antibiotic produced by *Bacillus licheniformis* which has been shown to inhibit enzymatic dephosphorylation of C_55_P(P) in the presence of divalent cations (Stone and Strominger, 1971). To our surprise, Δ*dbcA*; vec is sensitive to bacitracin at high levels (Figure 6A) and overexpression of DbcA can also sensitize E264 to bacitracin (Supplementary Figure S4). We cloned two homologs of UppP/BacA from *B. thailandensis* (*BTH_I1512* and *BTH_I2750*) and overexpressed them individually in E264 and Δ*dbcA* strains to examine if they could complement bacitracin and colistin sensitivity of Δ*dbcA*. BTH_I1512 displays 45% amino acid identity with *E. coli* UppP/BacA and BTH_I2750 has 36% amino acid identity with *E. coli* UppP/BacA. *BTH_I1512* was able to partially complement bacitracin sensitivity of Δ*dbcA* with 0.001% rhamnose induction, but it was unable to complement colistin sensitivity of Δ*dbcA* (Figure 6B). Overexpression of *BTH_I1512* with 0.1% inducer was toxic (Figure 6B). Interestingly, BTH_I2750 was unable to complement bacitracin sensitivity but was able to partially complement colistin sensitivity of Δ*dbcA* (Figures 6B,C). To determine if this partial complementation of colistin sensitivity of Δ*dbcA* is due to increase in species 2, we analyzed lipid A with BTH_I2750 overexpressed in E264 and Δ*dbcA*. There was no increase in species 2 by BTH_I2750 with either 0.01 or 0.1% rhamnose induction (Figure 6D), suggesting that this partial complementation was independent of lipid A modification, and supporting a possible multifactorial mechanism of extreme colistin resistance in *Burkholderia* spp. (Loutet and Valvano, 2011).

## Discussion

The increasing threat of multidrug-resistant (MDR) bacterial pathogens with stagnant discovery of new class of effective antibiotics has led to renewed interest in reviving older antibiotics like colistin (polymyxin E), a polycationic cyclic polypeptide belonging to polymyxin family of antibiotics (Talbot et al., 2006). Despite its reported nephrotoxicity and neurotoxicity, colistin still remains the most reliably active agent against many MDR gram-negative bacterial infections (Koch-Weser et al., 1970; Falagas and Kasiakou, 2005; Li et al., 2005). Colistin has been classified as “Reserve Group” of antibiotics to be used as a last-resort antibiotic against MDR infections that cannot be treated by any other conventional antibiotics (Hsia et al., 2019). However, the efficacy of colistin has been challenged by emerging acquired colistin resistance in several human pathogens and the global spread of plasmid acquired colistin resistance (Liu et al., 2016; McGann et al., 2016; Rolain et al., 2016; Elbediwi et al., 2020; Marano et al., 2020). Interestingly, species like *Burkholderia* are intrinsically resistant to colistin and the resistance may be multifactorial (Loutet and Valvano, 2011; Hamad et al., 2012; Malott et al., 2012; El-Halfawy and Valvano, 2014). We have previously shown that DedA protein, DbcA is required for extreme colistin resistance in *B. thailandensis* (Panta et al., 2019). We had proposed a model that the lower membrane potential observed in Δ*dbcA* may cause defects in PMF dependent step(s) in lipid A modification. Here, we demonstrate that overexpression of *arn* transport genes can partially complement the colistin sensitivity of Δ*dbcA* by increasing the abundance of lipid A modified with two Ara4N (species 2). We also show that the lower membrane potential of Δ*dbcA* is corrected at pH 5.5 media compared to pH 7.5 and this correction of membrane potential at pH 5.5 is consistent with the increase of species 2 at pH 5.5, suggesting that the lipid A modification at pH 7.5 by DbcA is dependent on PMF. We show for the first time that DbcA is also required for bacitracin resistance. This is also the first study to show that the lipid A modification, maintenance of membrane potential, and the colistin resistance in *Burkholderia thailandensis* is all linked to the pH of the media.

One of the steps of lipid A modification includes flipping of undecaprenyl-P-Ara4N from the inner leaflet of the cytoplasmic membrane to outer leaflet, carried out by EmrE like transporters ArnEF in *E. coli* (Yan et al., 2007). The small multidrug resistance transporter EmrE uses PMF to efflux a broad range of cationic compounds (Morrison et al., 2015; Robinson et al., 2017; Hussey et al., 2020). The ArnEF homolog, *BTH_I2194* is present in an operon along with *BTH_I2196* and the ArnT homolog, *BTH_I2195* that we designate as the transport operon (T). The genes involved biosynthesis are present in a nearby operon that we designate as the biosynthesis operon (B). We constructed several conditional mutants (E264B, E264T Δ*dbcA*B, and Δ*dbcA*T) by genomic insertion of an inducible rhamnose promoter in front of either *arn* “B” operon or *arn* “T” operon (Figure 1A). This allowed us to control the expression of these operons by the addition of rhamnose in the media.

The death of all the conditional mutants (E264B, E264T Δ*dbcA*B, and Δ*dbcA*T) when grown without rhamnose (Figure 1B) is consistent with the essentiality of *arn* genes in *B. thailandensis*, and as reported for *B. cenocepacia* (Ortega et al., 2007; Hamad et al., 2012) and also consistent with unavailability of transposon insertion mutants of *arn* genes (Gallagher et al., 2013). Slight growth of E264T without rhamnose (Figures 1B,C) might arise from residual expression of *arn* transport operon since the rhamnose promoter appears to be leaky in several of our experiments (data not shown). Next, we looked at the lipid A profile for all our conditional mutants along with E264 and Δ*dbcA*. Since Ara4N modification is an essential process, we were unable to measure lipid A in the complete absence of rhamnose in either strain. The level of lipid A species 2 increased with increasing amount of rhamnose in Δ*dbcA*B, and Δ*dbcA*T (Figure 2B). The amount of species 2 for Δ*dbcA*B was less compared to Δ*dbcA*T even with the same amount of rhamnose induction (Figure 2B). It is possible that overexpression of the transport operon might have stepped up the already PMF deficient transport step of lipid A modification in Δ*dbcA.* It should also be noted that species 2 in wild type E264 strains were largely independent of rhamnose concentration, suggesting efficient modification even at very low expression levels. It is also possible that there might be unknown post-transcriptional regulation or energy requiring steps facilitating such an efficient modification of lipid A in E264. We propose that this unknown factor might be PMF, which might be acting as a limiting factor in lipid A modification process.

To examine if this increase in species 2 is enough to complement colistin sensitivity of Δ*dbcA*, we performed a spot assay with varying colistin and rhamnose concentrations. We observed that overexpression of *arn* operons can partially complement colistin sensitivity of Δ*dbcA* (Figure 3). It is possible that lower PMF observed in Δ*dbcA* is limiting proper lipid A modification. Inefficient modification of lipid A in Δ*dbcA* is probably one of the effects of perturbed PMF, but there may be additional effects of this mutation that impact colistin resistance. Disturbed PMF in Δ*dbcA* might also have negative impacts on other PMF dependent efflux pumps, or PMF dependent processes that might be involved in colistin resistance directly or indirectly. Colistin resistance of *B. thailandensis* is defined by a complex synergistic relationship between active efflux and outer membrane diffusion (Krishnamoorthy et al., 2017).

Based on our previous findings in *B. thailandensis* and *E. coli*, we proposed that DedA proteins might be proton dependent transporters (Kumar and Doerrler, 2014, 2015; Kumar et al., 2016; Panta et al., 2019), suggesting that lower membrane potential observed in Δ*dbcA* might be caused by a defect in proton influx. We first examined whether artificially increasing proton influx by simply acidifying the media can compensate colistin sensitivity of Δ*dbcA*. The colistin MIC for Δ*dbcA* increased as the pH of the media decreased (Figure 4A, upper). In fact, the colistin resistance in E264 also depends on the pH of the media. Extreme alkaline pH of 8.0 can sensitize E264 to colistin (Figure 4A, lower), suggesting that colistin resistance in *B. thailandensis* is linked to pH homeostasis. This is the first study to our knowledge that establishes such a link and emphasizes a need to understand more about alkaline pH homeostasis in *B. thailandensis*. Why does alkaline pH sensitize Δ*dbcA* and E264 to colistin? We analyzed lipid A profiles at different pH. Interestingly, we found that lipid A modification in Δ*dbcA* is dependent on pH of the media. Higher the pH of the media, lower the amount of species 2 in Δ*dbcA* suggesting that lipid A modification with Ara4N is dependent on pH of the media in Δ*dbcA.* (Figure 4B). However, the lipid A modification was similar for E264 at all pH’s (Figure 4B), suggesting that DbcA is responsible in lipid A modification with Ara4N at higher pH’s. Lipid A analysis at pH 8 could not be done since the mutant strain underwent growth arrest under these conditions. Surprisingly, we found that lower membrane potential of Δ*dbcA* was corrected at pH 5.5 (Figure 4C), suggesting that the lipid A modification at pH 7.5 is PMF dependent. In addition, the membrane potential of E264 turned out to be significantly lower at pH 7.5 than that in pH 5.5 (Figure 4C). This suggests that increasing the pH of the media increases the possibility of E264 to have lower membrane potential and this might be the reason for lower colistin MIC in E264 at pH 8.0. This is further supported by our previous finding that lowering the membrane potential in E264 by CCCP also decreases the colistin MIC in E264 (Panta et al., 2019).

Why Δ*dbcA* has lower membrane potential at pH 7.5 compared to pH 5.5 remains an important question. We have observed that Δ*dbcA* is sensitive to divalent cations (Mg^++^ and Ca^++^), but not sensitive to monovalent cations (Na^+^ and K^+^) (Supplementary Figures S5A,B). The sensitivity of Δ*dbcA* to divalent cations can also be compensated by acidic pH (Supplementary Figure S5C). The presence of additional Mg^++^ and Ca^++^ in MH2 broth also decreased the colistin MIC of Δ*dbcA* (∼6 μg/ml colistin) compared to Mueller Hinton broth without added Mg^++^ and Ca^++^ (MIC ∼128 μg/ml colistin) (Supplementary Figure S5D). These observations suggest that DbcA is involved in divalent cations homeostasis, either directly by coupling proton movement to antiport of calcium or magnesium, or indirectly by other yet unidentified mechanisms. We have previously reported that overexpression of *mdfA*, a Na^+^-K^+^/H^+^ antiporter of the major facilitator superfamily essential for alkaline tolerance in *E. coli* (Lewinson et al., 2004), can compensate cell division defects and drug sensitivity of an *E. coli* strain lacking two partially redundant DedA family genes *yqjA* and *yghB* (Kumar and Doerrler, 2014). In *E. coli and S. aureus*, alkaline pH was shown to induce calcium influx and cytoplasmic calcium accumulation compared to acidic pH (Naseem et al., 2008; Nava et al., 2020). In light of these studies; it is possible that lower membrane potential of Δ*dbcA* at pH 7.5 is related to inefficient efflux of divalent cations at slightly alkaline pH.

The repression of lipid A modification through two component systems such as PhoPQ (Garcia Vescovi et al., 1996) by Ca^++^ and Mg^++^ could decrease the colistin MIC. Regulation of lipid A modifications in *B. thailandensis* is still largely unknown. The repression of lipid A modification by Ca^++^ and Mg^++^, however, cannot explain why Δ*dbcA* is sensitive to these divalent cations. Complementation of Ca^++^ and Mg^++^ toxicity in Δ*dbcA* by acidic media also could be due to increased lipid A modification with Ara4N. It is possible that reduced Ara4N modified lipid A in Δ*dbcA* at pH 7.0 increases Ca^++^ or Mg^++^ influx causing toxicity or that lipid A modification with Ara4N protects against divalent cation toxicity in this species.

We also found that the acidic pH complementation of colistin sensitivity of Δ*dbcA* requires expression of *arn* operons (Figure 5B). The construction of *arn* conditional mutants allowed us to look at the role of lipid A modification with Ara4N at acidic pH. Here we show that restraining *arn* expression can sensitize *B. thailandensis* to extreme acidic pH (Figure 5A). Furthermore, *B. cenocepacia* ArnT suppressor mutant MH-55 is also sensitive to acidic pH (Figure 5C) compared to its parental strain K56-2. These observations suggest that the physiological role of lipid A modification by Ara4N might be acid tolerance. Several reports show that the majority of *Burkholderia* species could only be isolated from acidic environments, suggesting a sensitivity to naturally occurring alkaline environments (Curtis et al., 2002; Caballero-Mellado et al., 2004; Kaestli et al., 2009; Stopnisek et al., 2014).

The link between pH sensing and lipid A modification is critical for virulence of many species of bacteria. Maximal Salmonella PhoPQ-dependent gene expression in murine RAW264.7 macrophages is seen following acidification of the phagosome (Alpuche Aranda et al., 1992) and TLR-dependent phagosomal acidification is required for Salmonella’s ability to survive phagocytosis (Arpaia et al., 2011). The lipid A profile from *S. typhimurium* infecting RAW264.7 cells showed modifications with aminoarabinose (Ara4N), phosphoethanolamine (PEtN), 2-hydroxymyristate, and palmitate, which resembled with the lipid A profile of *S. typhimurium* grown in acidic and low Mg^++^ minimal media (Gibbons et al., 2005). *Burkholderia cenocepacia* ArnT suppressor mutant strain MH55, that we reported to be acid sensitive, has been shown to cause reduced pathogenicity in both *Arabidopsis thaliana* and *Galleria mellonella* insect larvae without impacting the hosts’ ability to recognize pathogens (Khodai-Kalaki et al., 2015). Our study further supports the notion that lipid A modification with Ara4N is not only important for acidic tolerance, but also important for intracellular pathogens to establish infections, survive in acidic compartments of host immune cells, and evade host defenses.

Bacitracin sensitivity of Δ*dbcA* is intriguing, although the sensitivity is only observed at higher concentrations. *E. coli* bacitracin resistance and undecaprenyl pyrophosphate [C_55_P(P)] phosphatase have been shown to be conferred by two different classes of enzymes, either by UppP/BacA (Cain et al., 1993) or by YbjG, lpxT, pgpB, and BrcC_EC_ (all belonging to the phosphatidic acid phosphatase 2 (PAP2) superfamily of enzymes) (Harel et al., 1999; El Ghachi et al., 2005). Undecaprenyl-P has been proposed to flip back to the cytoplasm via UppP/BacA, which displays some amino acid identity to drug efflux pump MdfA, and other secondary transporters known to utilize PMF for their activity (El Ghachi et al., 2018; Workman et al., 2018). It is therefore possible that there is a PMF-dependent step during recycling of the bacterial carrier lipid C_55_P(P). If so, then Δ*dbcA* mutation might also cause defect in C_55_P(P) recycling and may result in colistin sensitivity. We found that one of the UppP/BacA homologs of *B. thailandensis*, BTH_I2750, can partially compensate colistin sensitivity of Δ*dbcA*, independent of lipid A modification, supporting the notion that extreme colistin resistance of *B. thailandensis* is multifactorial and more complex than we thought. One of the members of PAP2 superfamily in *H. pylori*, HupA was shown to be involved in UppP activity and polymyxin B resistance independent of lipid A modification (Gasiorowski et al., 2019). Plasmid-mediated colistin resistance determinants *mcr*-1 and *mcr*-3 were found to be linked to PAP2 containing gene *hpap*2 (Anyanwu et al., 2020; Gallardo et al., 2020). More studies need to be conducted to further explore possible role of C_55_P(P) recycling in colistin resistance in *Burkholderia* spp. Whether DbcA has any direct or indirect link in C_55_P(P) recycling also needs further investigation. The DedA protein family consists of highly conserved membrane proteins whose precise function remains a mystery. Here we show that DbcA is involved in PMF-dependent lipid A modification with Ara4N, which appears to be a major factor for maintaining extreme colistin resistance in *Burkholderia thailandensis*, E264.

## Data Availability Statement

The original contributions presented in the study are included in the article/Supplementary Material, further inquiries can be directed to the corresponding author/s.

## Author Contributions

WD and PP: conception and design of the study, the acquisition, analysis, and interpretation of the data, and writing of the manuscript. Both authors contributed to the article and approved the submitted version.

## Conflict of Interest

The authors declare that the research was conducted in the absence of any commercial or financial relationships that could be construed as a potential conflict of interest.
