# Supplementary material for: A Burkholderia thailandensis DedA Family Membrane Protein Is Required for Proton Motive Force Dependent Lipid A Modification
Source: Front Microbiol. 2021 Jan 12;11:618389. doi: 10.3389/fmicb.2020.618389 (PMC7835334; doi:10.3389/fmicb.2020.618389)
Supplement: Supplementary file 1 [file Data_Sheet_1.pdf]

Supplementary Material

A *Burkholderia thailandensis* DedA family membrane protein is required for proton motive force dependent lipid A modification

Pradip R. Panta and William T. Doerrler*

Department of Biological Sciences, Louisiana State University, Baton Rouge, LA, USA

*** Correspondence:**
William T. Doerrler; wdoerr@lsu.edu

Pradip R. Panta; ppanta1@lsu.edu

Keywords: colistin; pH and antibiotic resistance; lipopolysaccharide; membrane protein; proton motive force**,** lipid A modification and pH

Table S1. Bacterial strains and plasmids used in this study.

**Strains**	**Description**	**Source or Reference**
XL1 Blue	*recA*1 *endA1 gyrA96 thi-1 hsdR17 supE44 relA1 lac [F′ proAB lacIqZΔM15 Tn10* (Tet^R)	Stratagene
E. coli RHO3	SM10(lambda *pir*), kan^S; Δ*asd::FRT* Δ*aphA::FRT*	(Lopez et al., 2009)
E264	Wild-type *Burkholderia thailandensis*	(Brett et al., 1998)
Δ*dbcA*::FRT	E264 Δ*dbcA::FRT*	(Panta et al., 2019)
E264;vec	E264 transformed with pSCrhaB2 Tmp^R	(Panta et al., 2019)
Δ*dbcA*;vec	Δ*dbcA*::FRT transformed with pSCrhaB2 Tmp^R	(Panta et al., 2019)
E264;*dbcA*	E264 transformed with pSC*dbcA*	(Panta et al., 2019)
Δ*dbcA*;*dbcA*	Δ*dbcA*::FRT transformed with pSC*dbcA*	(Panta et al., 2019)
E264B	E264-P*rhaB*::*arnB-BTH_I2189*	This study
E264T	E264-P*rhaB*::*BTH_2196-BTH_I2194*	This study
Δ*dbcA*B	Δ*dbcA*B-P*rhaB*::*arnB-BTH_I2189*	This study
Δ*dbcA*T	Δ*dbcA*B-P*rhaB*::*BTH_2196-BTH_I2194*	This study
**Plasmids**		
pSCrhaB2	Expression vector; ori$_{pBBR1}$*rhaR, rhaS,* P_{rhaB}Tmp^R*mob*+	(Cardona and Valvano, 2005)
pSC*dbcA*	pSCrhaB2 expressing *dbcA* with His$_6$ tag at C terminus	(Panta et al., 2019)

pSCuppP1	pSCrhaB2 expressing BTH_I1512 with His$_6$ tag at C terminus	This study
pSCuppP2	pSCrhaB2 expressing BTH_I2750 with His$_6$ tag at C terminus	This study
pSC200	Tmp^R mob$^+$ Ori$_{R6K}$ *rhaR rhaA* P_{rhaB}	(Ortega et al., 2007)
pSC200Ara4NB	pSC200 cloned with 290bp portion of *BTH_I2193* (*arnB*)	This study
pSC200Ara4NT	pSC200 cloned with 330 bp portion of *BTH_I2196*	This study

Table S2. Oligonucleotide primers used in this study.

Primer name	Primer Sequence (5' → 3')
FWAra4NB	ATATAACATATGAGCCAGACTTCCGTTCCTTTC
REVAra4NB	ATATAATCTAGACGTCTCGAGGATCACGTTGCTC
FWAra4NT	ATATATCATATGCACCAACCCGGCC
REVAra4NT	ATATAATCTAGAGTTCGAGCAGCATCGCGAAG
ConfirmAra4NBFW	CGAAATAGTAATCACGAGGTCAGGTTC
ConfirmAra4NTFW	CGAAATAGTAATCACGAGGTCAGGTTC
ConfirmAra4NBREV	CCTTCGATCGACGTGATGTTCTTG
ConfirmAra4NTREV	CTGGAAATCCATCGTCTCGACG

FWBTH_I2750	ATATATACATATGATGAGCCTCTGGTTTCTGGTATTTCTG
REVBTH_I2750	ATATATAAGCTTGAAATGCCAATGGCGGCAGATCAAAC
FWBTH_I1512	ATATTACATATGATGGACTGGATACTGATTTGCAAAGC
REVBTH_I1512	ATATATAAGCTTTCAGATCCACTCGATCCAGCCGCTATAC
ConfirmpSCrhaB2FW	CATCATCACGTTCATCTTTCCCTG
ConfirmpSCrhaB2REV	GCAAATTCTGTTTTATCAGACCGC

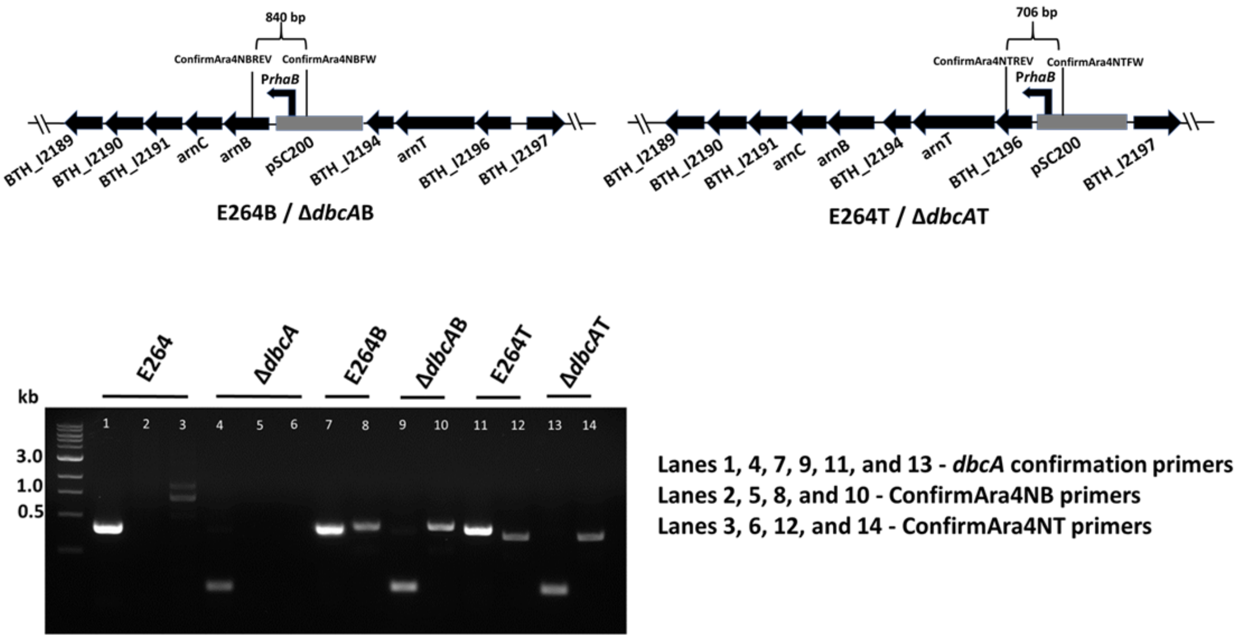

Figure S1. PCR confirmation of E264B/Δ*dbcA*B and E264T/Δ*dbcA*T strains. Listed primers were used to confirm *dbcA* deletion from E264B/Δ*dbcA*B and E264T/Δ*dbcA*T strains.

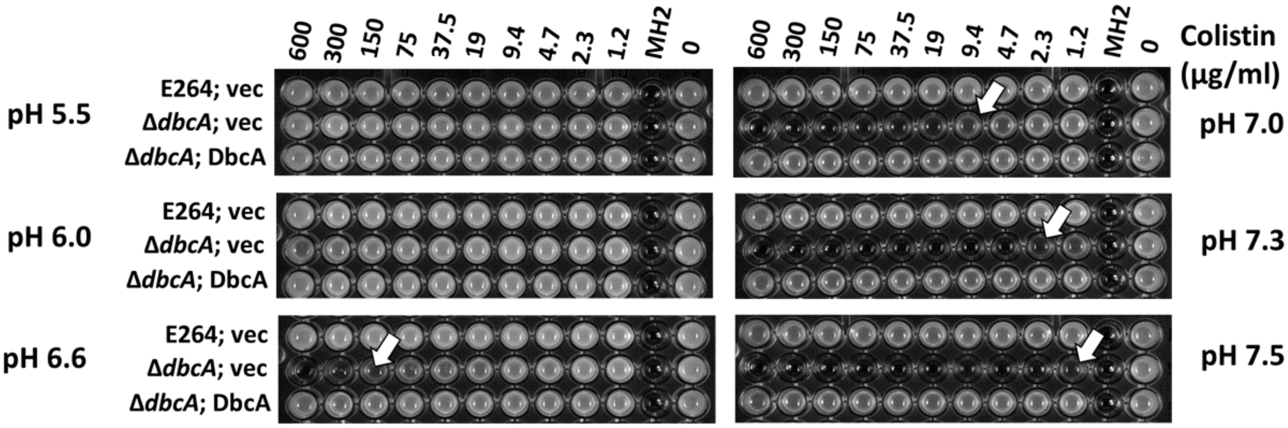

Figure S2. Minimal inhibitory concentration (MIC) using broth microdilution method. 1:100 dilutions from $3x10^8$ cells of indicated strains were inoculated in MH2 media at different pH with different amounts of colistin as indicated. 100mM MES was used for pH 5.5, 6.0, and 6.6 media. 70mM BTP was used for pH 7.0, 7.3, and 7.5. Plates were grown at 37ºC and analyzed after 24 hours. Approximate MICs are denoted by white arrows.

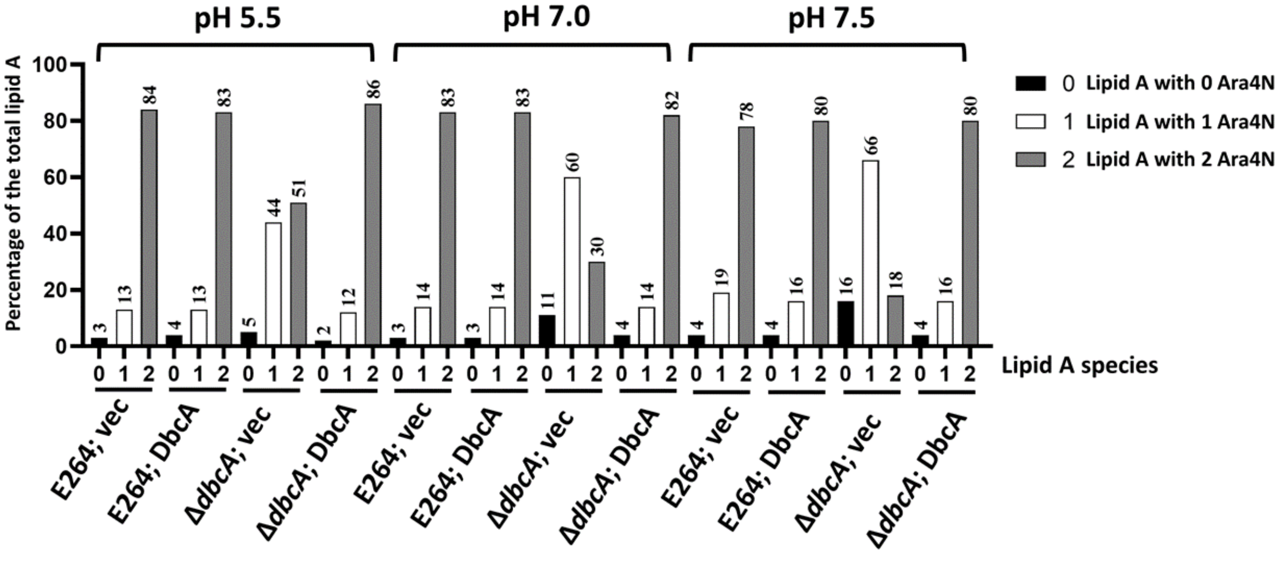

Figure S3. Percentage of the total lipid A for Figure 4B. Quantification as described in Materials and Methods.

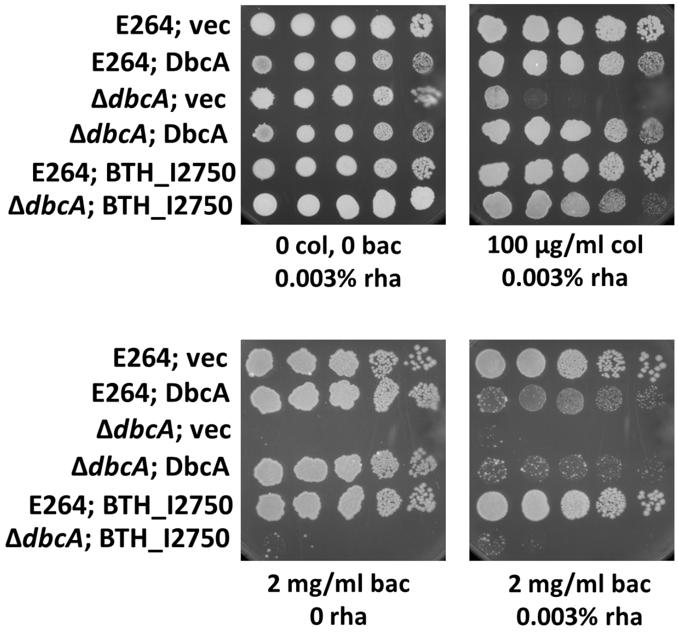

Figure S4. Overexpression of *dbcA* with 0.003% rhamnose is toxic to E264. 10-fold dilutions of indicated strains were spotted on LB containing 100 µg/ml Tmp, with 100 µg/ml of colistin or 2 mg/ml bacitracin and with or without 0.003% rhamnose. Plates were analyzed after 48 hours at 37°C.

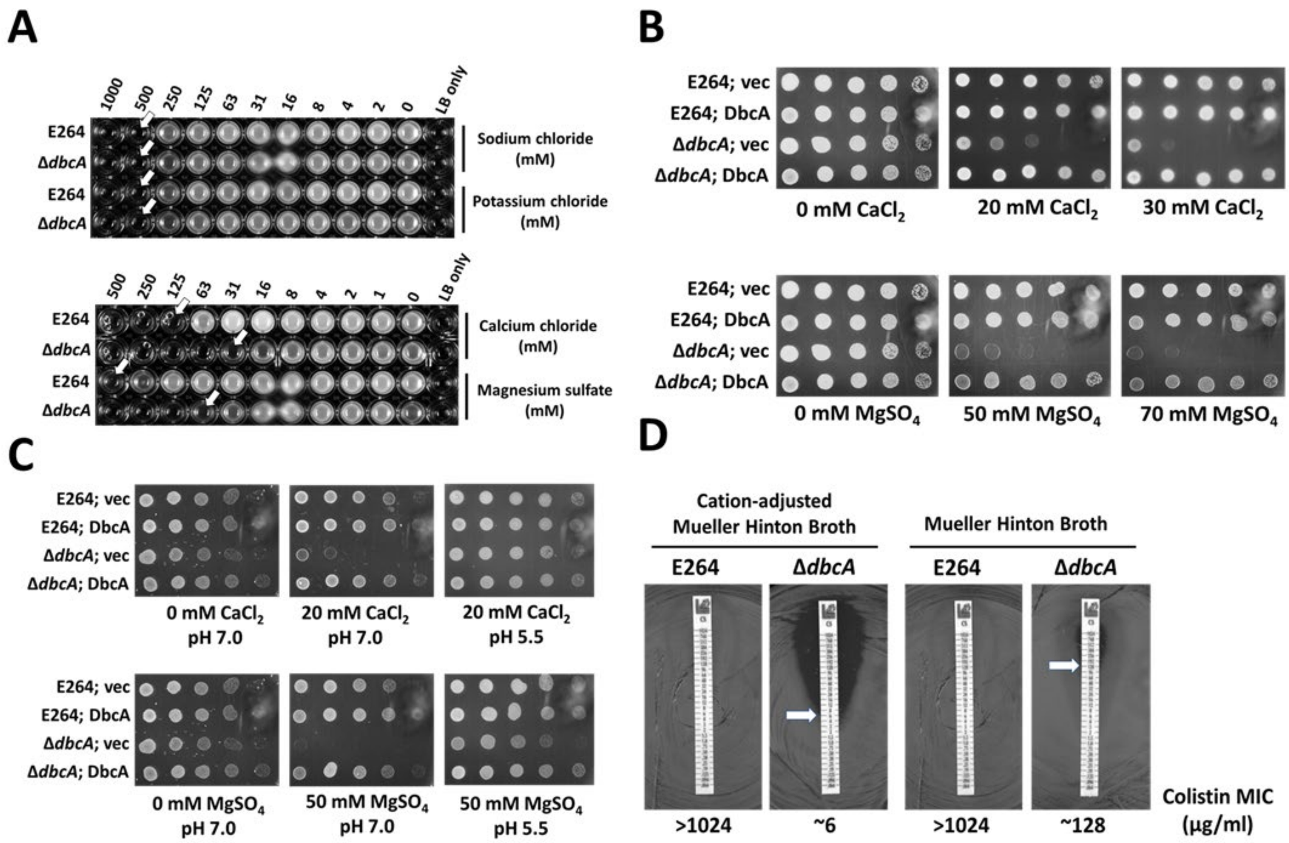

Figure S5. Sensitivity of Δ*dbcA* to Ca^{++} and Mg^{++}. **(A)** MIC for different cations using broth microdilution method for E264 and Δ*dbcA*. Approximate MICs are denoted by white arrows. **(B)** Sensitivity of Δ*dbcA* to Ca^{++} and Mg^{++} on solid media. 1:10 dilutions of indicated strains were spotted on LB agar media with 100 Tmp, 0.002% rhamnose, and different concentrations of calcium chloride ($CaCl_2$) and magnesium sulfate ($MgSO_4$) as indicated. **(C)** Complementation of Ca^{++} and Mg^{++} sensitivity of Δ*dbcA* by acidic pH media. 100mM Tris was used for pH 7.0 and 100mM MES was used for pH 5.5. **(D)** MIC using colistin E-test strips for E264 and Δ*dbcA* on two different types of media plates. Approximate MICs are denoted by white arrows. Cation-adjusted Mueller Hinton Broth contains ~20-25 mg/l Ca^{++} and 10-12.5 mg/l Mg^{++}, whereas Mueller Hinton broth does not have added Ca^{++} and Mg^{++} salts. pH for both media were adjusted to 7.3 using NaOH and HCl to exclude the pH effect for this experiment.

References:

Brett, P.J., DeShazer, D., and Woods, D.E. (1998). Burkholderia thailandensis sp. nov., a Burkholderia pseudomallei-like species. *Int J Syst Bacteriol* 48 Pt 1**,** 317-320. doi: 10.1099/00207713-48-1-317.

Cardona, S.T., and Valvano, M.A. (2005). An expression vector containing a rhamnose-inducible promoter provides tightly regulated gene expression in *Burkholderia cenocepacia*. *Plasmid* 54(3)**,** 219-228. doi: 10.1016/j.plasmid.2005.03.004.

Lopez, C.M., Rholl, D.A., Trunck, L.A., and Schweizer, H.P. (2009). Versatile dual-technology system for markerless allele replacement in Burkholderia pseudomallei. *Appl Environ Microbiol* 75(20)**,** 6496-6503. doi: 10.1128/AEM.01669-09.

Ortega, X.P., Cardona, S.T., Brown, A.R., Loutet, S.A., Flannagan, R.S., Campopiano, D.J., et al. (2007). A putative gene cluster for aminoarabinose biosynthesis is essential for *Burkholderia cenocepacia* viability. *J Bacteriol* 189(9)**,** 3639-3644. doi: 10.1128/JB.00153-07.

Panta, P.R., Kumar, S., Stafford, C.F., Billiot, C.E., Douglass, M.V., Herrera, C.M., et al. (2019). A DedA Family Membrane Protein Is Required for Burkholderia thailandensis Colistin Resistance. *Frontiers in Microbiology* 10(2532). doi: 10.3389/fmicb.2019.02532.
